# Three DUF1996 Proteins Localize in Vacuoles and Function in Fungal Responses to Multiple Stresses and Metal Ions

**DOI:** 10.1038/srep20566

**Published:** 2016-02-03

**Authors:** Sen-Miao Tong, Ying Chen, Sheng-Hua Ying, Ming-Guang Feng

**Affiliations:** 1 Institute of Microbiology, College of Life Sciences, Zhejiang University, Hangzhou, Zhejiang, 310058, People’s Republic of China

## Abstract

Many annotated fungal genomes harbour high proportions of hypothetical proteins with or without domains of unknown function (DUF). Here, three novel proteins (342−497 amino acids), each containing only a single large DUF1996 (231−250 residues) region with highly conserved head (DPIXXP) and tail (HXDXXXGW) signatures, were expressed as eGFP-tagged fusion proteins and shown to specifically localize in the vacuoles of *Beauveria bassiana*, a filamentous fungal entomopathogen; therefore, these proteins were named vacuole-localized proteins (VLPs). The VLPs have one to three homologues in other entomopathogenic or non-entomopathogenic filamentous fungi but no homologues in yeasts. The large DUF1996 regions can be formulated as D-X_4_-P-X_5–6_-H-X-H-X_3_-G-X_25–26_-D-X-S-X-YW-X-P-X_123–203_-CP-X_39–48_-H-X-D-X_3_-GW; the identical residues likely involve in a proton antiport system for intracellular homeostasis. Single deletions of three VLP-coding genes (*vlp1*–*3*) increased fungal sensitivities to cell wall perturbation, high osmolarity, oxidation, and several metal ions. Conidial thermotolerance decreased by ~11% in two Δ*vlp* mutants, and UV-B resistance decreased by 41−57% in three Δ*vlp* mutants. All the changes were restored by targeted gene complementation. However, the deletions did not influence fungal growth, conidiation, virulence or Cu^2+^ sensitivity. Our findings unveiled a role for the DUF1996 regions of three *B. bassiana* VLPs in the regulation of multiple stress responses and environmental adaptation.

A large number of novel proteins with domains of unknown function (DUFs) exist in annotated genomes across a wide range of organisms and environments. A naming scheme for DUFs was established by adding DUF1 and DUF2 to the SMART database in the 1990s^1^. So-named proteins are now categorized and enumerated as DUFX, where X is numbered from 11 to 3,779 in the Pfam database although the numbering is not completely consecutive^2^. Most DUFs are from 30 to 100 residues long^3^,^4^. Increasing numbers of DUF protein families have been added to the Pfam database due to a lack of structural and functional information in the scientific literature as newly sequenced genomes are annotated. Thus, a great challenge in the postgenomic era is to characterize the functions of DUF protein families and update their information in international databases^5^,^6^.

Functional characterization of DUF protein families has been progressing since DUF1 and DUF2, which are widely distributed bacterial signalling proteins, were identified as GGDEF and EAL domains, respectively^1^. This progress has accelerated in the past few years, shedding light upon many DUF proteins from prokaryotes and eukaryotes. The DUF of a protein (YP_001302112.1) secreted by human intestinal microbes has been shown to exhibit an affinity for Ca^2+7^. A member of the DUF3068 family has been identified as a pore-forming protein that is associated with the passage of hydrophilic solutes in *Corynebacterium amycolatum*^8^. DUF2233, present in microbial proteins and mammalian transmembrane glycoproteins, has been proven to be essential for the function of the host protein as a phosphodiester glycosidase^9^. A DUF1565 protein (LIC11207) in pathogenic *Leptospira* strains has been shown to delay polymorphonuclear cell apoptosis and to exert significant antigenicity against human convalescent sera^10^. Lp95, a leptospiral DUF1554 protein from *Escherichia coli*, can bind extracellular matrix components and activate E-selectin on the endothelial cells of human umbilical veins^11^. DUF1605 in the helicase DHX9 acts as a MyD88-dependent DNA sensor in the cytosol of plasmacytoid dendritic cells and hence is vital for C-phosphate-G binding, suggesting a role for this helicase in viral sensing^12^. DUF1943 and DUF1944 in the proteins of vertebrates and invertebrates aid the function of vitellogenin as a pattern recognition receptor and opsonin^13^. Apart from these advances, more DUF proteins have been characterized in model plants, such as *Arabidopsis*. AtGXMT1, previously classified as a member of the DUF579 family, has been shown to be required for the 4-*O*-methylation of glucuronic acid and hence to play an important role in the modulation of biopolymer interactions in the plant cell wall^14^. DUF581 proteins have been identified as potential mediators conferring tissue- and stimulus-type specific differences in the regulation of a SNF1-related kinase that responds to the availability of carbohydrates and environmental stresses^15^. Three RING domain-containing E3 Ub ligases with C-terminal DUF1177 domains were confirmed to regulate drought resistance in *Arabidopsis*^16^. Expression and functional analyses have revealed the sensitivity of two DUF642 family genes to L-galactono-1,4-lactone, the terminal precursor for ascorbic acid, thus contributing to *Arabidopsis* development^17^. Two DUF579 proteins (IRX15 and IRX15-L) have been proven to function in xylan synthesis in *Arabidopsis*^18^. The expression of DUF1618 family genes varies in rice cultivars and in response to stress or hormone conditions, suggesting a link between this protein family and rice development and fitness^19^. In addition, DUF3444 is associated with highly conserved catalytic motifs that are characteristic of the methyltransferases required for genome-wide methylation in the overall development of *Physcomitrella patens*^20^.

A large number of DUF or hypothetical proteins in human, animal and plant pathogenic fungi remain generally uncharacterized. For example, up to 1000 proteins are functionally unknown in *Saccharomyces cerevisiae*, comprising 17% of the annotated genes in this model yeast genome^21^. Large numbers of DUF families in the increasing genomes of fungal pathogens also pose a great challenge for understanding molecular mechanisms involved in host infection and environmental adaptation. *Beauveria bassiana* is a filamentous fungal insect pathogen and a source of many fungal insecticides against arthropod pests^22^. This insect pathogen usually lacks a sexual form and has the broadest host range among fungal pathogens known to date^23^,^24^. Approximately 25% of the genes in the annotated genome of *B. bassiana*^25^ encode hypothetical proteins. Such unknown proteins are likely involved in the fungal adaptation to diverse hosts and habitats. Therefore, the purpose of this study was to characterize three novel proteins in the DUF1996 family. All three localized in vacuoles and played significant roles in the response of *B. bassiana* to multiple stresses and metal ions. This is the first report on subcellular localization and function of fungal DUF1996 family proteins.

## Results

### Sequence features of the DUF1996 family proteins discovered in *B. bassiana* and other fungi

**Figure 1:**
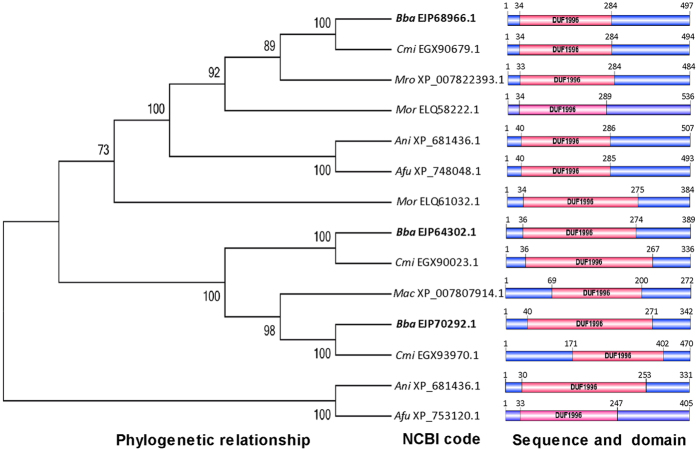
Phylogenetic and structural features of DUF1996 family homologues in different filamentous fungi. The NCBI accession code of each homologue follows the abbreviation of the fungal name (*Ani, Aspergillus nidulans*; *Afu, Aspergillus fumigatus*; *Bba, Beauveria bassiana*; *Cmi, Cordyceps militaris*; *Mac, Metarhizium acridum*; *Mro, Metarhizium robertsii*; *Mor, Magnaporthe oryzae*).

A BLAST search of the *B. bassiana* genome^25^ for WSC (cell Wall integrity and Stress response Component) domain-containing proteins resulted in a list of putative proteins containing one to five WSC domains. In this list, however, three proteins (NCBI codes: EJP70292.1, EJP68966.1 and EJP64302.1) had no WSC domain and hence were misannotated. This misannotation was also present in homologues in the genomes of *Cordyceps militaris*^26^, *Metarhizium robertsii* (previously sorted to *M. anisopliae* sensu lato) and *M. acridum*^27^, three other filamentous fungal insect pathogens. In contrast, homologues in the NCBI databases for *Aspergillus nidulans, A. fumigatus* and *Magnaporthe oryzae* were annotated as “hypothetical proteins”. All of the homologues in the aforementioned fungi had only a single DUF1996 domain, which was often present in the N-terminal region but occasionally in the central or even C-terminal region (Fig. 1). The DUF1996 proteins in *B. bassiana* were relatively closer to those in *C. militaris* and two *Metarhizium* species than to those of non-entomopathogenic fungi. However, such homologues were absent in several yeasts, including *S. cerevisiae*, based on the BLAST search.

The sequences of 14 DUF1996-containing proteins from filamentous fungi consisted of 272–536 amino acids, and their DUF1996 regions encompassed 47.6–68.8% of the total sequence lengths. None of these proteins contained meaningful motifs or signal peptides, as predicted with available bioinformatic software. Interestingly, all 14 DUF1996 regions shared highly conserved N-terminal (DXXXXP) and C-terminal (HXDXXXGW) motifs and could be formulated as D-X_4_-P-X_5–6_-H-X-H-X_3_-G-X_25–26_-D-X-S-X-YW-X-P-X_123–203_-CP-X_39–48_-H-X-D-X_3_-GW, based on sequence alignment analysis (Fig. S1). The domains of the DUF1996 homologues from all four fungal entomopathogens shared an even more conserved N-terminal region (DPIXXP) and contained many more identical residues (Fig. S2). It is worth noting that in the formula, the most variable region is located between proline 2 and cysteine 1, followed by regions encompassing proline 3 to histidine 3 and glycine 1 to aspartic acid 2. These variable regions were closely related to the domain or protein size.

### Subcellular localization and transcriptional expression of the three DUF1996 proteins in *B. bassiana*

**Figure 2:**
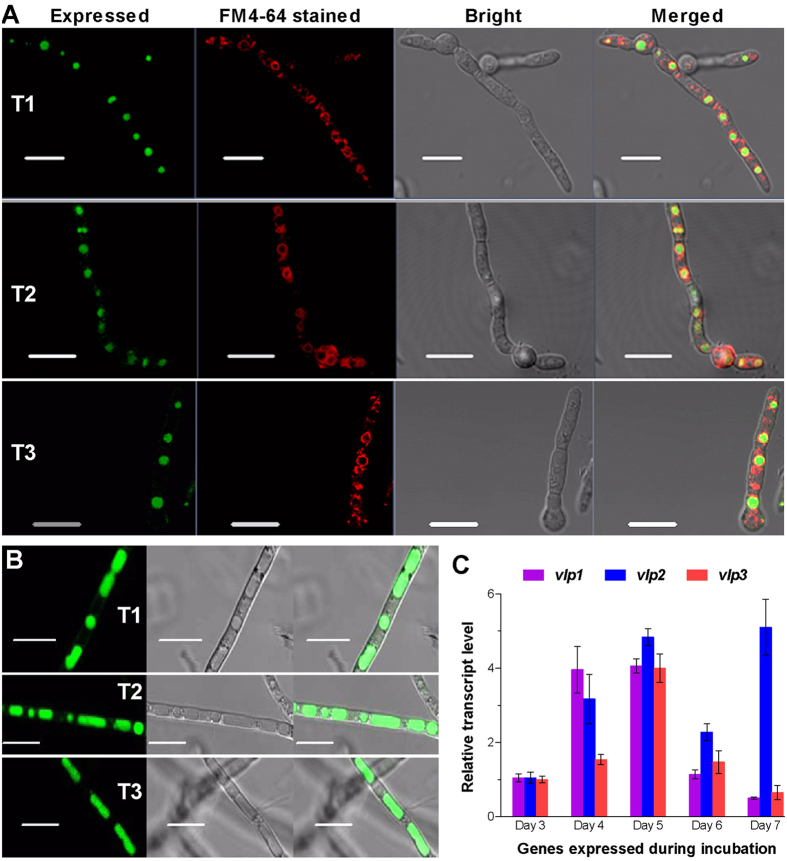
Subcellular localization and transcriptional profiles of the three DUF1996 proteins in *B. bassiana*. (**A**) Microscopic images for the eGFP-tagged fusions (green) expressed in the hyphae of transgenic strains (T1, T2 and T3) collected from 2-day-old SDB cultures and stained with membrane-specific FM4–64 (red). Note that the expressed green in spherical vacuoles is well defined by, but not merged with, the colour of the stained vacuolar membrane. (**B**) Microscopic images of the increased expression of the eGFP-tagged fusions in the tubular vacuoles of transgenic hyphae from 5-day-old SDB cultures. Scale bars: 10 μm. (**C**) Transcript levels of three vacuole-localized protein genes (*vlp1–3*) in wild-type *B. bassiana* throughout standard cultivation relative to day 3. Total RNA was extracted daily from an SDAY culture initiated by spreading 100 μl of a conidial suspension per plate. Error bars: SD from four independent cDNA samples analyzed via qRT-PCR with paired primers (Table S1).

To probe their subcellular localizations, each of the DUF1996 proteins was fused to an eGFP tag and expressed in the wild-type strain *B. bassiana* ARSEF 2860, resulting in the transgenic strains T1, T2, and T3. As shown by fluorescence microscopy, each eGFP-tagged fusion target protein (green) accumulated only in the spherical vacuoles of young hyphae (which were stained with FM4-64, a membrane-specific dye); these hyphae arose directly from the extension of germ tubes in 2-day-old Sabouraud dextrose broth (SDB) cultures (Fig. 2A). The accumulated green signals were well defined with respect to the stained membrane (red), but the two colours did not merge to form orange on the vacuolar membrane. The accumulation of the eGFP-tagged protein increased in the tubular vacuoles of aging hyphae from 5-day-old SDB cultures (Fig. 2B). Thus, the subcellular localization of the three *B. bassiana* DUF1996 proteins indicates that they are definitely vacuole-localized proteins (VLPs).

The VLP-coding genes *vlp1, vlp2* and *vlp3* (tag loci: BBA_01161, BBA_02001 and BBA_06684, respectively) were constitutively expressed in the WT strain over 7 days of standard cultivation in rich SDAY (Sabouraud dextrose agar plus yeast extract) at an optimal 25 °C (Fig. 2C). The transcripts of these genes peaked on day 5 but *vlp2* also showed another peak on day 7. Apparently, the transcriptional peak of each *vlp* gene occurred at approximately the same time as when the presence of the corresponding eGFP-tagged fusion protein in the tubular vacuoles peaked.

### Single *vlp* deletions increased cellular sensitivities to stress cues and metal ions

**Figure 3:**
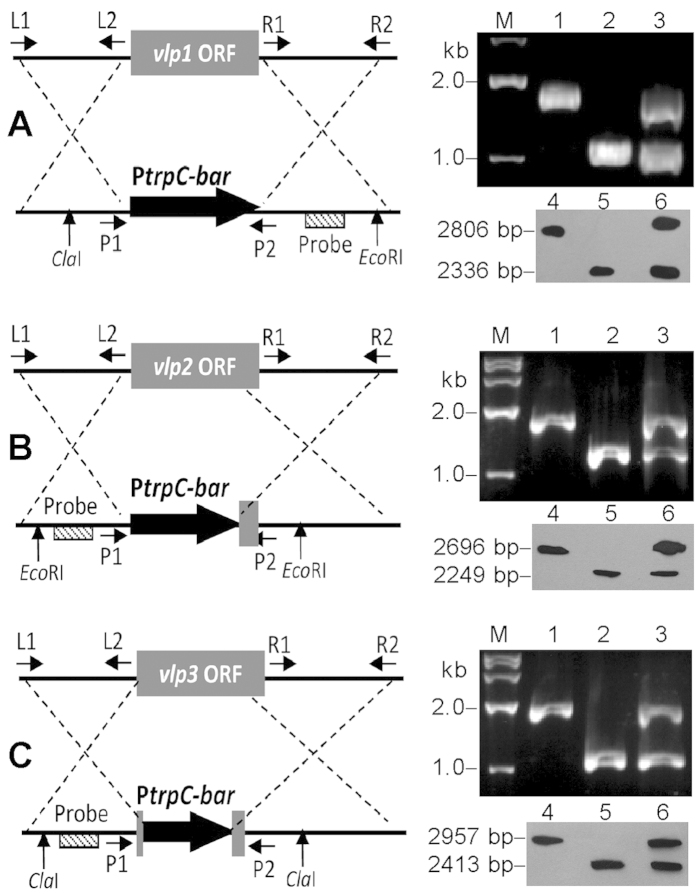
Generation and identification of *B. bassiana vlp* mutants. (**A–C**) Diagrams for the deletion of *vlp1, vlp2* and *vlp3* from WT, respectively, and their mutants identified by PCR (lanes 1**–**3) and Southern blotting (lanes 4**–**6) analyses with paired primers and amplified probes (Table S1). Lanes 1 and 4: WT. Lanes 2 and 5: Δ*vlp* mutant. Lanes 3 and 6: Δ*vlp::vlp* mutant. The genomic DNAs of the *vlp1, vlp2* and *vlp3* mutants used in Southern blotting hybridization were digested with *Cla*I/*Eco*RI, *Eco*RI/*Eco*RI and *Cla*I/*Cla*I, respectively.

Each of the three *vlp* genes was deleted from the WT by replacing its coding sequence (containing the entire domain region) with the *bar* marker, complemented by ectopically integrating a cassette with the full-length sequence and a *sur* marker in each deletion mutant (as described in Methods), and confirmed by PCR and Southern blotting analyses (Fig. 3). As a result, the three deletion mutants grew as well as the control strains (the parental WT and the complemented mutants) in rich SDAY or in minimal Czapek agar (CZA). Each strain was spotted onto plates (1 μl of a 10^6^-conidia/ml suspension per plate), and the resulting mean ( ± SD) colony diameter of each strain was approximately 31.9 ± 0.9 mm in SDAY (*F*_6,14_ = 1.74, *P* = 0.18) or 21.0 ± 0.8 mm in CZA (*F*_6,14_ = 1.65, *P* = 0.21) after 8 days of incubation at 25 °C. Conidial yields from the 7-day-old SDAY cultures, which were initiated by spreading 100 μl of 10^7^ conidia/ml suspension per plate, were not significantly different among the Δ*vlp* mutants and two control strains (Tukey’s HSD, *P* > 0.05). The median germination time (GT_50_), i.e., the time required for 50% germination of conidia at 25 °C, was 9.5 ± 0.3 h for all the tested strains (*F*_6,14_ = 1.69, *P* = 0.20). These data indicated no significant role for each *vlp* in fungal growth, conidiation or germination.

**Figure 4:**
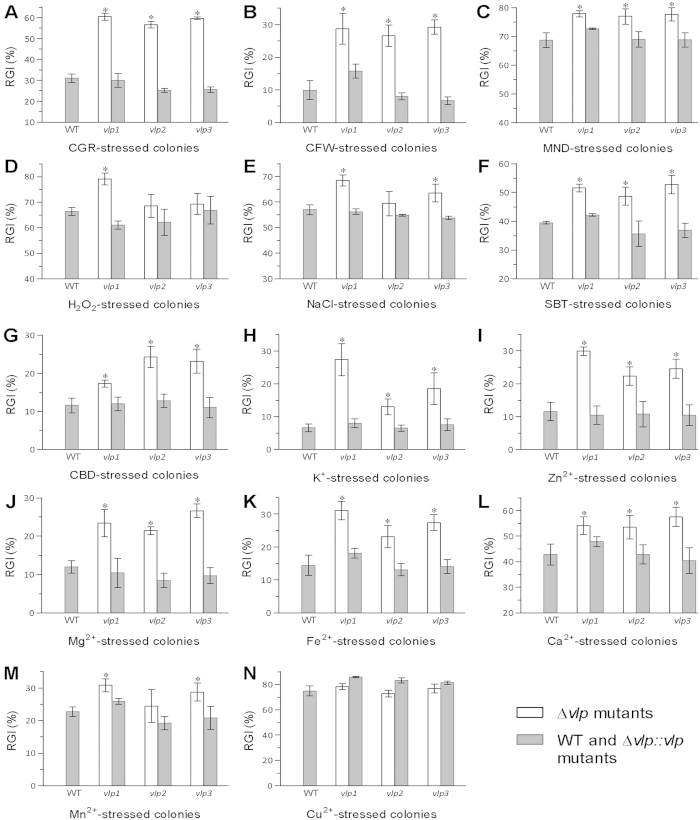
Single deletions of *B. bassiana vlp1–3* resulted in reduced multi-stress tolerance. (**A–G**) Relative growth inhibition (RGI) of fungal colony growth by CGR (Congo red 6 μg/ml), CFW (calcofluor white 0.5 mg/ml), MND (menadione 0.04 mM), H_2_O_2_ (2 mM), NaCl (0.4 M), SBT (sorbitol 1 M), or CBD (carbendazim 0.1 μg/ml) added to CZA, respectively. (**H**–**N**) RGI values of fungal strains in response to K^+^ (5 mM KCl), Zn^2+^ (3 mM ZnCl_2_), Mg^2+^ (3 mM MgCl_2_), Fe^2+^ (1 mM FeSO_4_), Ca^2+^ (0.1 M CaCl_2_), Mn^2+^ (3 mM MnCl_2_) or Cu^2+^ (2 mM CuCl_2_), respectively. All CZA colonies were initiated by spotting 1 μl of a conidial suspension per plate, followed by an 8-day incubation at 25 °C. Asterisked bars in each graph differ significantly from unmarked bars (Tukey’s HSD, *P* < 0.05). Error bars: SD from three replicates.

All the deletion mutants were more sensitive to various chemical stresses and metal ions than the control strains during 8-day colony growth initiated by spotting 1-μl aliquots of conidial suspensions onto CZA plates. Co-cultivation with Congo red (6 μg/ml) and calcofluor white (0.5 mg/ml) led to 26−29% (Fig. 4A) and 17−19% (Fig. 4B) growth suppression, respectively, compared with the mean size of the WT colonies. Compared with WT growth, the growth of each deletion mutant was also significantly (7−13%) more suppressed by co-cultivation with menadione (0.04 mM), H_2_O_2_ (2 mM), NaCl (0.4 M), sorbitol (1 M) and carbendazim (0.1 μg/ml) (Fig. 4C–G), with the exception of Δ*vlp2*, which exhibited an insignificant change (Tukey’s HSD, *P* > 0.05) in sensitivity to NaCl and H_2_O_2_, and Δ*vlp3*, which was not significantly more sensitive to H_2_O_2_.

Additionally, the three Δ*vlp* mutants were significantly more sensitive to five or six metal ions than WT (Tukey’s HSD, *P* < 0.05). As illustrated in Fig. 4H–M, the sensitivities of the mutant strains to K^+^ (5 mM), Zn^2+^ (3 mM), Mg^2+^ (3 mM), Fe^2+^ (1 mM) and Ca^2+^ (0.1 M) were increased by 6.4−20.7%, 22.3−29.9%, 9.5−14.7%, 8.6−16.7% and 10.7−14.7%, respectively. An increased sensitivity to all five metal ions was minimal for Δ*vlp2* and maximal for Δ*vlp1*. In contrast, Δ*vlp3* was most sensitive to Ca^2+^. Δ*vlp1* and Δ*vlp3* were also significantly more sensitive to Mn^2+^ (3 mM). However, all three Δ*vlp* mutants and the control strains were equally sensitive to 2 mM Cu^2+^ (Fig. 4N). All changes in cellular sensitivities to stressful chemicals and metal ions were restored by targeted gene complementation.

### Single *vlp* deletions reduced conidial thermotolerance and UV-B resistance but did not affect virulence

**Figure 5:**
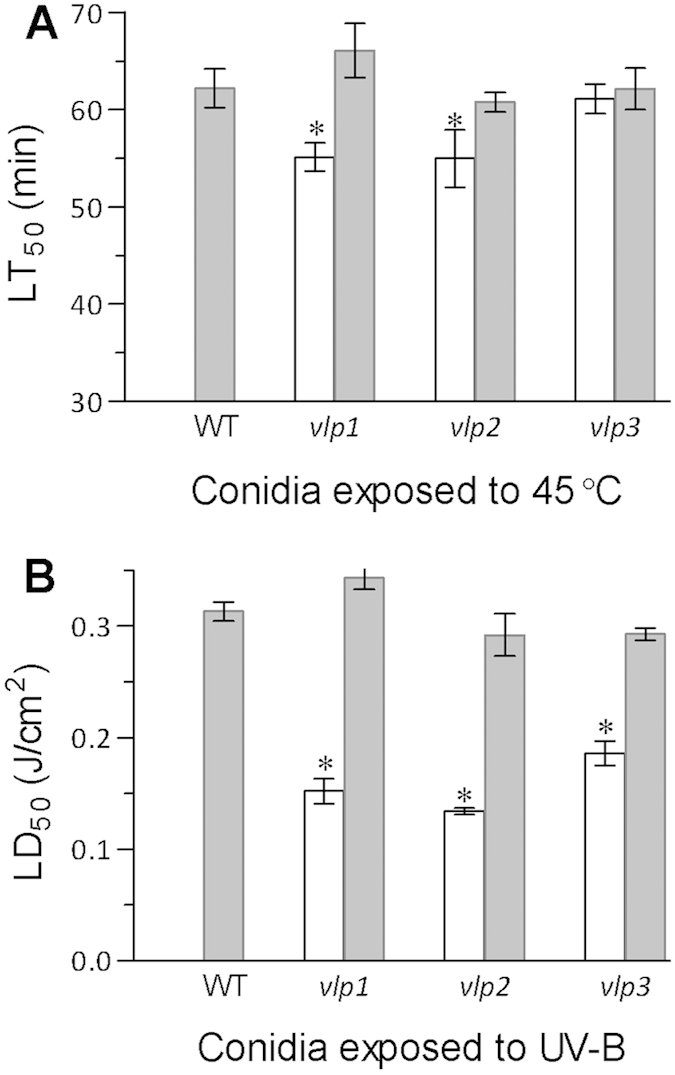
Single deletions of *vlp1–3* reduced the biological control potential of *B. bassiana*. (**A**) LT_50_ (min) for conidial tolerance to wet-heat stress at 45 °C. (**B**) LD_50_ (J/cm^2^) for conidial tolerance to UV-B irradiation. Asterisked bars in each graph differ significantly from unmarked bars (Tukey’s HSD, *P* < 0.05). Error bars: SD from three replicates.

Because conidial thermotolerance and UV-B resistance influence the pathogenic potential of fungi against arthropod pests, these phenotypes were quantified in each Δ*vlp* mutant via modelling analyses. The median lethal time (LT_50_) was measured in a gradient wet-heat assay at 45 °C to quantify conidial thermotolerance, and the median lethal dose (LD_50_) was determined for conidia exposed to UV-B irradiation. As a result, conidial LT_50_ values were significantly shortened by ~11% in both Δ*vlp1* and Δ*vlp2*, but not in Δ*vlp3*, versus the WT (Fig. 5A). Conidial LD_50_ values were reduced by 51% in Δ*vlp1*, 57% in Δ*vlp2* and 41% in Δ*vlp3* compared with that of the WT (Fig. 5B). These two phenotypic changes were restored in all the complemented mutants.

In standardized bioassays, however, all the Δ*vlp* mutants and control strains exhibited similar virulence against *Galleria mellonella* larvae. The LT_50_ values of all strains fell in a narrow range of 5.3−5.7 days when a suspension of 10^7^ conidia/ml was topically applied to induce normal cuticular infection (*F*_6,14_ = 1.42, *P* = 0.27); similarly, 5 μl of a 10^5^ conidia/ml suspension of each strain was injected into larval haemocoels to induce cuticle-bypassing infections (*F*_6,14_ = 0.26, *P* = 0.96), which resulted in LT_50_ values of 5.2−5.5 days.

## Discussion

The three DUF1996-containing proteins of *B. bassiana* characterized in this study feature extremely large DUF1996 domains—231−250 residues long (which is far beyond the upper limit of 100 residues for DUFs in other organisms^3^,^4^)—and have one to three homologues in other entomopathogenic or non-entomopathogenic fungi. The DUF1996 regions of these homologues are also 214−251 residues long, except for the smallest domain (130 residues) present in the smallest homologue in *M. acridum* (272 amino acids). Of note, all 14 DUF1996 homologues in different filamentous fungi do not contain any other domain, despite being annotated as hypothetical proteins or WSC domain-containing proteins, and their domain sequences can be described by the same formula regardless of their respective lengths. Therefore, the three proteins in *B. bassiana* and their homologues in other fungal entomopathogens should be annotated as hypothetical proteins, as were their homologues in non-entomopathogenic fungi, because they all belong to the DUF1996 family.

Moreover, all three *B. bassiana* DUF1996 proteins are localized only in the vacuoles, as shown by the expression of the eGFP-tagged fusion proteins in the WT. This subcellular localization confirms that these proteins are not putative WSC domain-containing proteins at all because authentic WSC proteins, such as Wsc1–4 in *S. cerevisiae* and WscA/B in *A. nidulans*, are localized in the plasma membrane and may act as sensors upstream of the cell-wall integrity pathway^28^,^29^,^30^,^31^. Therefore, we named these DUF1996 proteins as vacuole-localized proteins *Vlp1, Vlp2* and *Vlp3*. This naming scheme will likely suit other fungal DUF1996 homologues if they are localized in vacuoles. However, more experimental evidence is required to properly rename the entire DUF1996 family.

More importantly, the vacuolar localization of the three proteins is in agreement with their roles in the cellular events of *B. bassiana*. As illustrated by multi-phenotypic analyses of single-gene deletion mutants in parallel with control strains, these proteins functioned in almost all cellular responses to cell-wall perturbation, high osmolarity, oxidation, fungicidal stress and multiple metal ions although the increased sensitivity to each chemical stressor varied among their deletion mutants. Previously, some vacuole-localized proteins have been shown to play important roles in plant tolerance to stresses, although they are structurally distinct from the DUF1996 family. For instance, a vacuole-localized β-glucosidase contributes to *Arabidopsis* drought tolerance^32^, a stress associated with high osmolarity. The expression of a vacuole-localized BURP-domain protein from soybean enhances *Arabidopsis* tolerance to Cd^2+^ and Cu^2+^ 33. Fungal vacuoles are acidic storage compartments with certain similarities to plant vacuoles^34^ and play vital roles in the storage of Ca^2+^, phosphate and amino acids; pH and osmotic regulation; ion homeostasis; and cytoplasmic detoxification^35^,^36^. The uptake of many toxic ions by the vacuole for cytosol detoxification is energized by proton antiport systems for arginine, arginine-lysine, histidine, phenylalanine-tryptophan, tyrosine, glutamine-asparagine and isoleucine-leucine, thus enabling amino acid accumulation^37^. Most of the mentioned amino acids are present in the DUF1996 domains of the three VLPs, implicating that they may involve in the proton antiport system required for intracellular homeostasis in *B. bassiana*. Perhaps for this reason, our Δ*vlp* mutants were differentially less tolerant to all the tested chemicals and metal ions except Cu^2+^, indicating a disturbance in cellular homeostasis.

In addition, conidial tolerance to UV-B irradiation, solar irradiation that is harmful to fungal viability, was greatly impaired in our Δ*vlp* mutants. A significant loss of conidial thermotolerance also occurred in Δ*vlp1* and Δ*vlp2*. These results indicate that *Vlp1−3* are vital for fungal adaptation to outdoor adversity and hence contribute substantially to the biological control potential of *B. bassiana* against arthropod pests. However, these proteins are not relevant to virulence. Taken together, our results show that DUF1996 is a vital domain for the three VLPs in *B. bassiana*, like many repported DUFs that are essential in bacteria^38^.

## Methods

### Microbial strains and culture conditions

WT *B. bassiana* and mutants were grown at 25 °C in a light/dark cycle of 12/12 h on rich SDAY (4% glucose, 1% peptone and 1.5% agar plus 1% yeast extract) for normal growth or on minimal CZA (3% sucrose, 0.3% NaNO_3_, 0.1% K_2_HPO_4_, 0.05% KCl, 0.05% MgSO_4_ and 0.001% FeSO_4_) for phenotypic assays. *E. coli* Top10 and DH5α from Invitrogen (Shanghai, China) were cultured in Luria-Bertani medium plus kanamycin (100 μg/ml) or ampicillin (100 μg/ml) for plasmid propagation. *Agrobacterium tumefaciens* AGL-1, cultivated in YEB medium^39^, was used as a T-DNA donor for fungal transformation.

### Locating DUF1996 proteins in *B. bassiana* and other fungi

An online search with a WSC query (http://www.ncbi.nlm.nih.gov/guide/proteins/) was performed to locate “WSC domain-containing proteins” in the *B. bassiana* genome^25^. Among the putative WSC domain-containing proteins located in the fungal genome, three did not contain any WSC domains but did contain a single DUF1996 domain, as determined by further BLAST analysis (http://blast.st-va.ncbi.nlm.nih.gov/Blast.cgi). The sequences of the three DUF1996 proteins (now named *Vlp1, Vlp2* and *Vlp3*) were used as queries to search for homologues in the NCBI databases of three other filamentous entomopathogens (*C. militaris, M. robertsii*, and *M. acridum*) and some non-entomopathogenic, but representative, fungi (*A. nidulans, A. fumigatus, M. oryzae* and *S. cerevisiae*). The full sequences of all the DUF1996 homologues located in *B. bassiana* and the other fungi were analyzed to reveal possible signal peptides or referable motifs using the programs SignalP 3.0 server (http://www.cbs.dtu.dk/services/SignalP-3.0/), TMHMM Server v. 2.0 (http://www.cbs.dtu.dk/services/TMHMM/) and NetNGlyc 1.0 server (http://www.cbs.dtu.dk/services/NetNGlyc/), followed by phylogenetic analysis with the MEGA5 software^40^. Subsequently, their DUF1996 regions were aligned with DNAman 8.0 to reveal domain sequence identity.

### Transcriptional profiling and the subcellular localization of *Vlp1–3*

To gain temporal transcript profiles for each *vlp* in normal (standard) WT cultures, 100-μl aliquots of a 10^7^-conidia/ml suspension were evenly spread onto cellophane-overlaid SDAY plates and incubated for 7 days at 25 °C in a 12/12 h light/dark cycle. From day 3 onwards, total RNA was extracted daily from the SDAY cultures, prepared using RNAiso Plus Reagent (TaKaRa, Dalian, China) and reverse-transcribed into cDNAs using a PrimeScript RT reagent kit (TaKaRa). Each cDNA sample (20-fold dilution) was used as a template to quantify the transcript of each target gene via quantitative real-time PCR (qRT-PCR) with paired primers (Table S1). The 18S rRNA of *B. bassiana* was used as an internal standard. The relative transcript level of each gene was calculated as the ratio of its transcript in the daily cDNA sample over that on day 3 using the 2^−ΔΔCt^ method^41^. All the treatments included four cDNA samples as replicates.

The open reading frame (ORF) of each *vlp* gene was amplified from the WT cDNA with paired primers (Table S1) and fused to the 5’-end of *eGFP* tag pAN52- eGFP-bar^42^, yielding pAN52-vlp::eGFP-bar. All the plasmids were linearized with *PmeI/Eco*RI and then transformed into the WT as described previously^42^. Transgenic strains expressing the fusions *vlp1::eGFP, vip2::eGFP* and *vip3::eGFP* respectively were selected in terms of the *bar* resistance to phosphinothricin (200 µg/ml) and of the expressed eGFP (enhanced green fluorescence protein) under a fluorescent microscope. The conidia of positive transformants were suspended in SDB (i.e., agar-free SDAY), followed by shaking (150 rpm) cultivation at 25ºC. The resultant hyphae were stained with the membranes-specific dye FM4-64 (Sigma) and visualized in bright/fluorescent field of view under a confocal microscope. Microscopic images for the expressed eGFP (green) and the stained color (red) in the same field were collected and merged using an image browser to determine the subcellular localization of each target Vlp.

### Generation of *vlp* mutants

Our previous plasmids, p0380-bar and p0380-sur-gateway^42^, were used as backbones to construct deletion and complementation plasmids for each VLP gene. First, 5′ and 3′ fragments (~1500 bp each) comprising the partial coding and flanking regions of each gene were amplified from the WT with two pairs of primers (Table S1) and inserted into p0380-bar, yielding p0380-vlp*x*_up-bar-vlp*x*_dn (*x* = 1, 2 or 3) for targeted gene deletion. Second, the full-length sequence of each *vlp* gene (with flanking regions) was amplified from the WT and used to replace the gateway fragment in p0380-sur-gateway, resulting in p0380-sur-vlp*x*, for targeted gene complementation. Each *vlp* gene was deleted by transforming the deletion plasmid into WT, thus replacing all (*vlp1*) or most (*vpl2* and *vlp3*) of the coding sequence (containing the entire DUF1996 region) with the *bar* marker, and then complemented by ectopically integrating a cassette containing the full-length sequence and the *sur* marker via *Agrobacterium*-mediated transformation^39^ into the deletion mutant. Putative mutant colonies grown on selective medium were screened for *bar*-encoded resistance to phosphinothricin (200 μg/ml) or *sur*-encoded resistance to chlorimuron ethyl (10 μg/ml). Recombination events in the mutants were examined via PCR and Southern blot analyses with paired primers and amplified probes (Table S1). Positive deletion mutants were evaluated together with the control strains for phenotypic changes in triplicate experiments described below.

### Assessments of radial growth, conidiation capacity and germination rate

One-microlitre aliquots of a 10^6^-conidia/ml suspension were spotted centrally on SDAY and CZA plates. After 8 days of incubation at 25 °C with a 12/12 h light/dark cycle, all fungal colonies were cross-measured, yielding a mean colony diameter for each strain as an index of growth rate.

The conidiation capacity of each strain was quantified from the SDAY cultures, which were initiated by spreading 100 μl of a 10^7^-conidia/ml suspension per plate and incubated for 7 days at 25 °C with a 12/12 h light/dark cycle. Three culture plugs (5 mm in diameter) were cut from each plate using a cork borer. Conidia in each plug were released into 1 ml 0.02% Tween 80 thorough vibration, followed by filtration to remove hyphal debris. The concentration of conidia in the suspension was determined using a haemocytometer and converted to the number of conidia per cm^2^ culture.

Three 100-μl aliquots of a 10^6^-conidia/ml suspension were evenly spread onto germination medium plates (2% sucrose, 0.5% peptone and 1.5% agar), followed by a 24-h incubation at 25 °C. Germination percentage was assessed at 2-h intervals using three microscopic counts per plate. The median germination time (GT_50_) was estimated by fitting the germination trend over the time of incubation.

### Assays for cellular responses to stressful chemicals and metal ions

One-microlitre aliquots of a 10^6^-conidia/ml suspension were spotted onto CZA plates alone (control) or supplemented with: (1) Congo red (6 μg/ml) or calcofluor white (0.5 mg/ml) for cell-wall perturbation; (2) NaCl (0.4 M) or sorbitol (1 M) for osmotic stress; (3) menadione (0.04 mM) or H_2_O_2_ (2 mM) for oxidative stress; carbendazim (0.1 μg/ml) for fungicidal stress; and (4) K^+^ (5 mM KCl), Cu^2+^ (2 mM CuCl_2_), Mn^2+^ (3 mM MnCl_2_), Zn^2+^ (3 mM ZnCl_2_), Mg^2+^ (3 mM MgCl_2_), Ca^2+^ (0.1 M CaCl_2_) or Fe^2+^ (1 mM FeSO_4_) for metal ion stress. The resulting plates were incubated for 8 days at 25 °C in a 12/12 h light/dark cycle, followed by cross-measuring the diameter of each colony. The relative growth inhibition (RGI) of each chemical stressor or metal ion was computed as (*S*_c_−*S*_t_)/*S*_c_ × 100, where *S*_c_ and *S*_t_ are colony areas (mm^2^) from the control and the stress treatment of each strain, respectively.

### Assays for conidial thermotolerance, UV-B resistance and virulence

Aerial conidia from the SDAY cultures were assayed for thermotolerance and UV-B resistance by exposing samples to 45 °C wet heat for 0−120 min and UV-B irradiation (weighted wavelength: 312 nm) at gradient doses of 0−0.8 J/cm^2^, respectively, as described previously^43^. After exposure, each sample was incubated for 24 h under optimal conditions and then examined microscopically to estimate the germination percentage. The ratio of the germination percentage in a given heat or UV-B stress treatment over that in the unstressed control treatment was calculated as the relative survival index. The relative survival trend of each strain versus the intensities of the heat or UV-B stress was fitted to a survival equation, generating LT_50_ (min) for conidial thermotolerance or LD_50_ (J/cm^2^) for conidial UV-B resistance.

Fungal virulence to *G. mellonella* larvae (~300 mg *per capita*) obtained from a vendor (Da Mai Chong Insectaries, Wuxi, Jiangsu, China) was bioassayed by topical application and intrahaemocoel injection of conidial suspensions, respectively, as described elsewhere^43^. The resultant time-mortality trends were subjected to probit analysis, yielding an LT_50_ for each strain against the larvae.

### Statistical analysis

All the phenotypic observations from the triplicate experiments were subjected to one-factor (strain) analysis of variance, followed by Tukey’s honestly significant difference (HSD) test for the means of each phenotype between each deletion mutant and two control strains.

## Additional Information

**How to cite this article**: Tong, S.-M. *et al*. Three DUF1996 Proteins Localize in Vacuoles and Function in Fungal Responses to Multiple Stresses and Metal Ions. *Sci. Rep*. **6**, 20566; doi: 10.1038/srep20566 (2016).

## Supplementary Material

Supplementary Information
