# Supplementary material for: Three DUF1996 Proteins Localize in Vacuoles and Function in Fungal Responses to Multiple Stresses and Metal Ions
Source: Sci Rep. 2016 Feb 3;6:20566. doi: 10.1038/srep20566 (PMC4738358; doi:10.1038/srep20566)
Supplement: Supplementary Information [file srep20566-s1.pdf]

Scientific Reports

Supplementary Material

Three DUF1996 Proteins Localize in Vacuoles and Function in Fungal Responses to Multiple Stresses and Metal Ions

Sen-Miao Tong, Ying Chen, Sheng-Hua Ying, and Ming-Guang Feng*

Institute of Microbiology, College of Life Sciences, Zhejiang University, Hangzhou, Zhejiang, 310058, People’s Republic of China (*Corresponding to: mgfeng@zju.edu.cn)

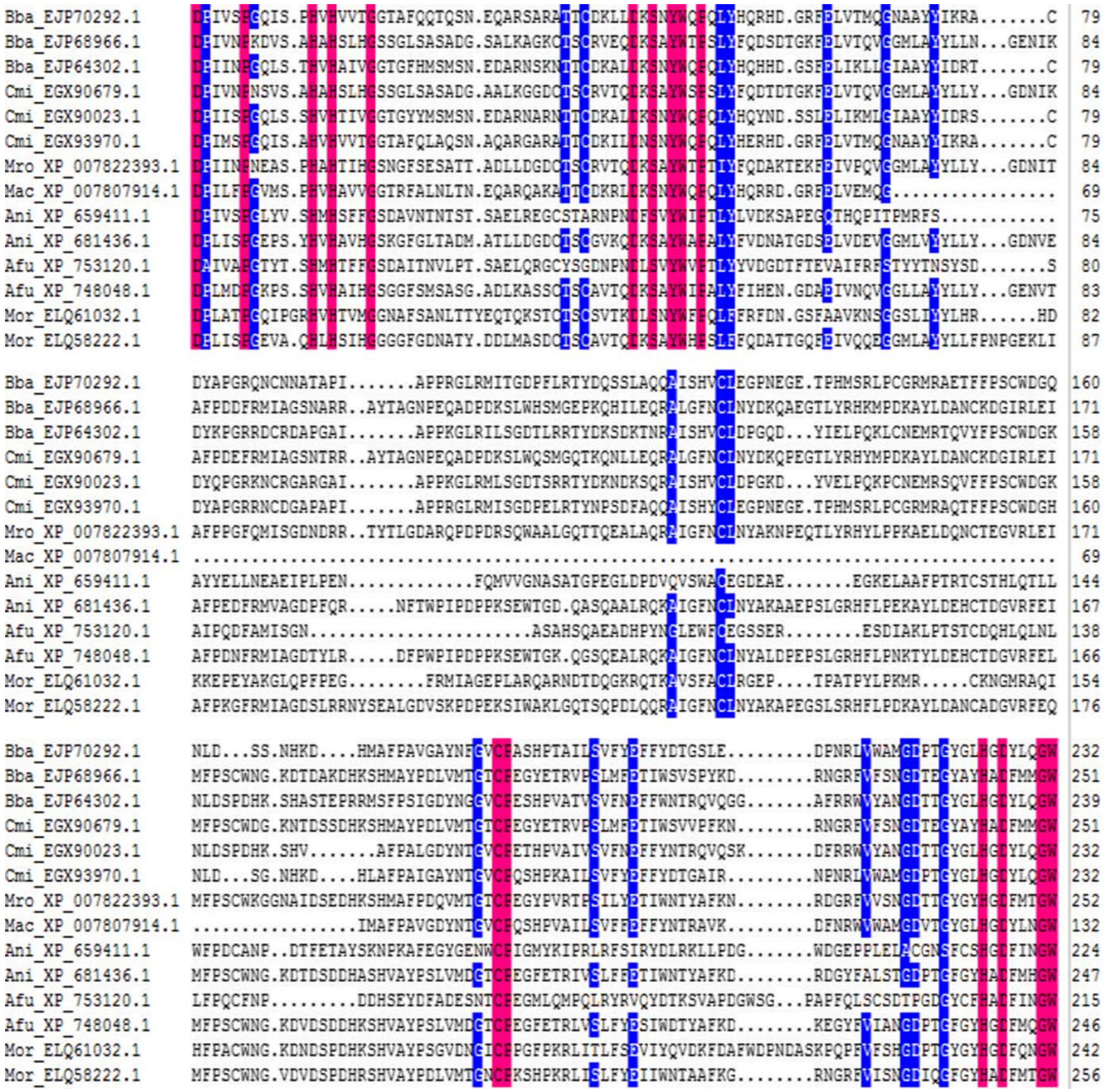

Figure S1 Sequence features of domain regions in the DUF1996 family proteins of several filamentous fungi. NCBI accession code of each protein follows the fungal name (Ani, *Aspergillus nidulans*; Afu, *Aspergillus fumigatus*; Bba, *Beauveria bassiana*; Cmi, *Cordyceps militaris*; Mac, *Metarhizium acridum*; Mro, *Metarhzium robertsii*; Mor, *Magnaporthe oryzae*). Red: 100% identity. Blue: 75% identity.

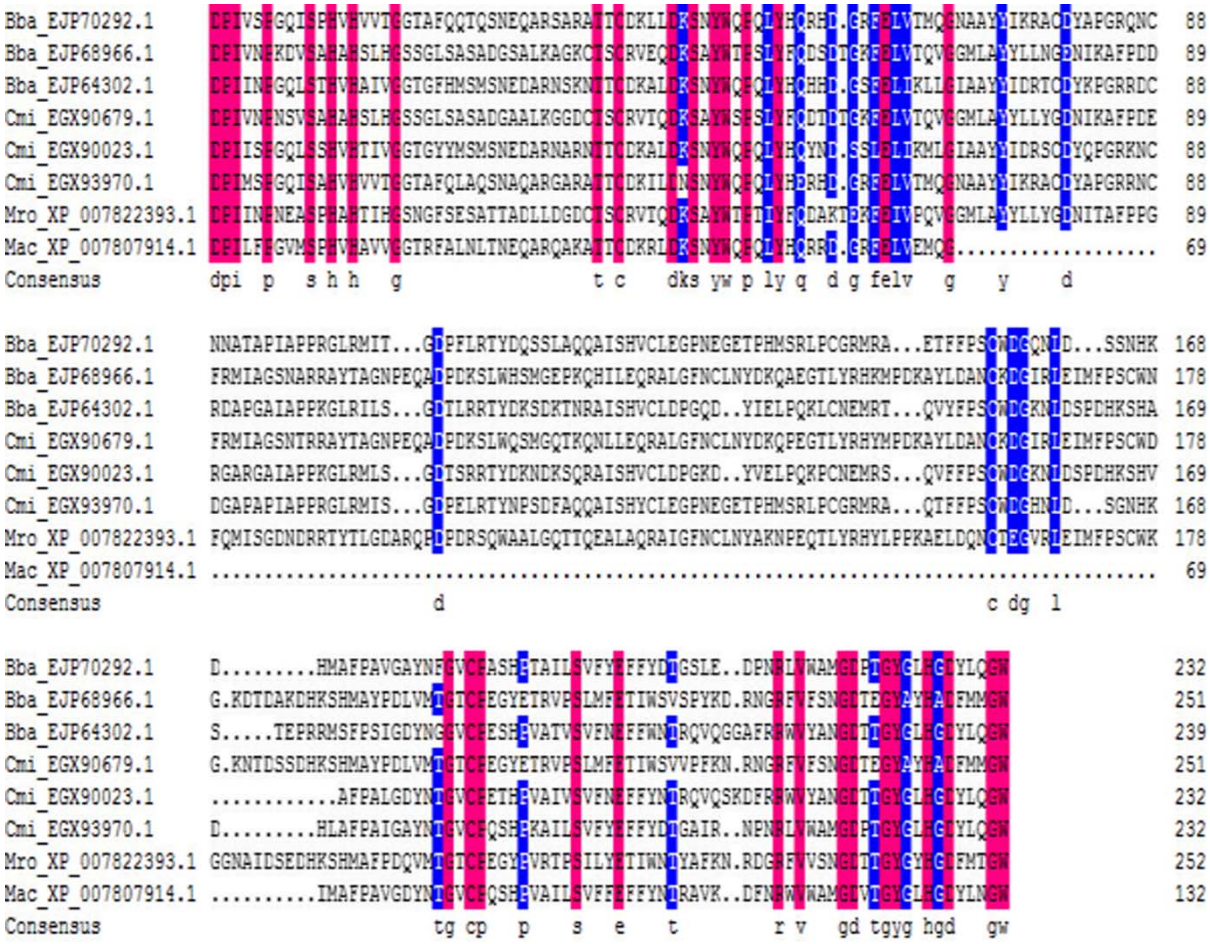

Figure S2 Sequence features of domain regions in the DUF1996 proteins of four filamentous fungal insect pathogens. NCBI accession code of each protein follows the fungal name (Bba, *Beauveria bassiana*; Cmi, *Cordyceps militaris*; Mac, *Metarhizium acridum*; Mro, *Metarhzium robertsii*). Red: 100% identity. Blue: 75% identity.

Table S1. Paired primers designed for manipulation of three VLP-coding genes (*vlp1–3*) in *B. bassiana* and identification of their deletion and complementary mutants.

**Primers**	**Sequences (5′–3′)***	**Purpose**
Vlp1-F/R	AGCTTTGTTTAAACATGCCCTCAAATTGGTCACTC / AAAAAGACTAGTTACCGGGTCCCCGGTAACC	Cloning *vlp1* cDNA
Vlp2-F/R	AGCTTTGTTTAAACATGTTCAAGCTTACTACCGCCGTG / AAAAAGACTAGTGCTCCGGCGGACGTGAGC	Cloning *vlp2* cDNA
Vlp3-F/R	AGCTTTGTTTAAACATGCTTTCTAAATACCTTGTTGC / AAAAAGACTAGTGAGAGCAACACAGCGTTGG	Cloning *vlp3* cDNA
Vlp1up-F/R	AAACCGGAATTCTCTCACCAATGAACCTTGTCTG / AAACCCAAGCTTCAAGAAGAAACGAAGAACGGAG	Cloning *vlp1* 5′(1501 bp)
Vlp1dn-F/R	AACTAGTCTAGAATGCTAAGCGACGGCAGAGAAT / AAAAAAGTTAACTTGCGGCTCTGAAAGGTGAAAT	Cloning *vlp1* 3′(1525 bp)
Vlp2up-F/R	AAACGCGGATCCCACCTACCTACCTAATCGTTGG / AAACCCAAGCTTTCTTTGATGGTAAGGAGTGGAC	Cloning *vlp2* 5′(1584bp)
Vlp2dn-F/R	AA CTAGTCTAGAACTCCTACCGCTTCTACCAACC / AAAGGAAGATCTCCAGCCAGCCCCTTTCCTTT	Cloning *vlp2* 3′(1588 bp)
Vlp3up-F/R	AAACCGGAATTCGCGAGTCTGGTCTTGAGCATT / AAACGCGGATCCAAGGTAGCAATGGGGGAGAG	Cloning *vlp3* 5′(1424 bp)
Vlp3dn-F/R	AACTAGACTAGTGGCATTTGAGGGAGAAGATTG / AAAAAAGTTAACGTCAGGCTCTTCTTCTGTTTGC	Cloning *vlp3* 3′(1637 bp)
Vlp1fl-F/R	*GGGGACAAGTTTGTACAAAAAAGCAGGCT*GAGGCTCCAACCCATAGACATT/*GGGGACCACTTTGTACAAGAAAGCTGGGT*CTTGACGGCGACCATCATTTAC	Cloning full-length *vlp1*
Vlp2fl-F/R	*GGGGACAAGTTTGTACAAAAAAGCAGGCT*TCCGTTGACCAGTGCGTGAGT/*GGGGACCACTTTGTACAAGAAAGCTGGGT*GAATGTGTTGCCTGGTGAGACG	Cloning full-length *vlp2*
Vlp3fl-F/R	*GGGGACAAGTTTGTACAAAAAAGCAGGCT*TAGAAAGGCAAATCCCTCCAT/*GGGGACCACTTTGTACAAGAAAGCTGGGT*TTATCCGTCACAAGTAGTCCGT	Cloning full-length *vlp3*
pVlp1- F/R	AAATCCTTGATACCGTCTCCGT / AAATCGCAGTATTCAGTGTCGC	PCR detection of *vlp1*
pVlp2- F/R	ACACCATCTCAGACTGCTCATC / CTGCTGCGTCCAGAGTATTTTG	PCR detection of *vlp2*
pVlp3- F/R	TACCTTGTTGCTCTCTCCCCCA / GCGTATCGGTTTTCGGCTTTTG	PCR detection of *vlp3*
sbVlp1-F/R	TTGTGCGACACTGAATACTGC / CATGGGATCTTCCGCTTCT	For Southern probe of *vlp1*
sbVlp2-F/R	TTCTTTCATTGGCGAGGAT / TGAGTGGCTGGTGAGTTGG	For Southern probe of *vlp2*
sbVlp3-F/R	ACAGCCAATGCCACCCTAC / TTGCCAAACCCTTCGGATA	For Southern probe of *vlp3*
qVlp1-F/R	GGCAATCCTGTCCGTGTTC / CAGGTAGTCGCCGTGGAG	qRT-PCR detecting *vlp1*
qVlp2-F/R	CCACTCCTACCGCTTCTAC / GTGTTGCCGTTGGTGATG	qRT-PCR detecting *vilp2*
qVlp3-F/R	GGAGGTTGGCTTGAATGG / ATGGCGTAGAATCTCTGTTG	qRT-PCR detecting *vlp3*
q18S-F/R	TGGTTTCTAGGACCGCCGTAA / CCTTGGCAAATGCTTTCGC	qRT-PCR of 18S rRNA

* Underlined regions: introduced cleavage sites of restriction enzymes for the deletion of *vlp1* (*Eco*RI/*Hin*dIII and *Xba*I/*Hpa*I), *vlp2* (*Bam*HI/*Hin*dIII and *Xba*I/*Bgl*II) and *vlp3* (*Eco*RI/*Bam*HI and *Spe*I/*Hpa*I). Italicized and underlined regions: the gateway exchange fragments for each *vlp* complementation.
